# Liver injury without liver failure in COVID-19 patients: how to explain, in some cases, elevated ammonia without hepatic decompensation

**DOI:** 10.1186/s13054-020-03088-x

**Published:** 2020-06-16

**Authors:** Patrick M. Honore, Leonel Barreto Gutierrez, Luc Kugener, Sebastien Redant, Rachid Attou, Andrea Gallerani, David De Bels

**Affiliations:** ICU Department, Centre Hospitalier Universitaire Brugmann University Hospital, Place Van Gehuchtenplein, 4, 1020 Brussels, Belgium

We read with great interest the recent research letter by Cardoso et al. who describe the liver injury seen with COVID-19 [1]. We would like to provide some additional information. In our large cohort of COVID-19 patients, we had several patients who did not regain consciousness as expected, even when sedation had been stopped for 4–5 days. Electroencephalogram (EEG) in these patients demonstrated a metabolic pattern. In the process of working through the differential diagnoses, we measured serum ammonia levels and were surprised to see that in two patients the ammonia level was elevated 3 times above the normal limit. While those patients had liver injury but absolutely no sign of liver failure, nor were they receiving medications that could explain hyperammonemia, such as valproate or amiodarone [2]. Both patients had experienced very severe diarrhea several days prior to admission. Baseline Glasgow Coma Score (GCS) was difficult to determine as both patients were intubated by an emergency team on site. CT scan of the brain was unremarkable. Both patients were treated with classical medical therapy including lactulose, but, despite increasing doses of lactulose for 3 days, ammonia levels remained unchanged. We decided that if there was no progress within 72 h, continuous renal replacement therapy (CRRT) would be started to remove ammonia. As the ammonia was below 200 mg/dL, there was no acute indication to start CRRT to avoid brain edema. We were surprised to see that both patients regained consciousness 48 and 72 h later respectively, and ammonia levels normalized. Retrospectively, we hypothesize that the pre-admission diarrhea may have resulted in secondary carnitine deficiency, as described in the literature [3], leading to hyperammonemia unresponsive to medical therapy [4]. CRRT dramatically reduces ammonia levels, but ultimately can worsen the situation by further reducing the level of carnitine [5]. As we did not measure serum carnitine levels and we did not supply the patients with carnitine supplementation, the diagnosis of carnitine deficiency in these cases remains only a hypothesis. Clinicians should keep this diagnosis in mind in COVID-19 patients with severe diarrhea.

## Data Availability

Not applicable.

## Abbreviations

EEG: Electroencephalogram
CRRT: Continuous renal replacement therapy
